# Combining Multi-Shell Diffusion with Conventional MRI Improves Molecular Diagnosis of Diffuse Gliomas with Deep Learning

**DOI:** 10.3390/cancers15020482

**Published:** 2023-01-12

**Authors:** Golestan Karami, Riccardo Pascuzzo, Matteo Figini, Cosimo Del Gratta, Hui Zhang, Alberto Bizzi

**Affiliations:** 1 Department of Neuroscience, Imaging and Clinical Sciences, Gabriele D’Annunzio University, 66100 Chieti, Italy; 2 Institute for Advanced Biomedical Technologies, Gabriele D’Annunzio University, 66100 Chieti, Italy; 3 Department of Neuroradiology, Fondazione IRCCS Istituto Neurologico Carlo Besta, 20133 Milan, Italy; 4 Centre for Medical Image Computing and Department of Computer Science, University College London, London WC1V 6LJ, UK

**Keywords:** adult-type gliomas, multi-shell diffusion MRI, molecular subtypes, IDH-mutation, 1p/19q codeletion, deep learning

## Abstract

**Simple Summary:**

The integration of advanced magnetic resonance imaging (MRI) has the potential to enable the improved prediction of the molecular diagnosis of adult-type gliomas. In this context, this study investigated whether deep learning-based predictive models can benefit from adding multi-shell diffusion MRI to conventional MRI. We evaluated the performance of an exemplar deep learning model for differentiating (1) isocitrate dehydrogenase (IDH)-mutation versus IDH-wildtype; (2) 1p/19q codeletion versus 1p/19q non-codeletion; and (3) IDH-mutation with or without 1p/19q codeletion, and IDH-wildtype. The model achieved the best prediction performance in our cohort of 146 patients in all three tasks when multi-shell diffusion MRI and conventional MRI are combined. These results demonstrate the specific added value provided by advanced diffusion MRI, extending the current literature on building deep learning models based on multiple MRI modalities.

**Abstract:**

The WHO classification since 2016 confirms the importance of integrating molecular diagnosis for prognosis and treatment decisions of adult-type diffuse gliomas. This motivates the development of non-invasive diagnostic methods, in particular MRI, to predict molecular subtypes of gliomas before surgery. At present, this development has been focused on deep-learning (DL)-based predictive models, mainly with conventional MRI (cMRI), despite recent studies suggesting multi-shell diffusion MRI (dMRI) offers complementary information to cMRI for molecular subtyping. The aim of this work is to evaluate the potential benefit of combining cMRI and multi-shell dMRI in DL-based models. A model implemented with deep residual neural networks was chosen as an illustrative example. Using a dataset of 146 patients with gliomas (from grade 2 to 4), the model was trained and evaluated, with nested cross-validation, on pre-operative cMRI, multi-shell dMRI, and a combination of the two for the following classification tasks: (i) IDH-mutation; (ii) 1p/19q-codeletion; and (iii) three molecular subtypes according to WHO 2021. The results from a subset of 100 patients with lower grades gliomas (2 and 3 according to WHO 2016) demonstrated that combining cMRI and multi-shell dMRI enabled the best performance in predicting IDH mutation and 1p/19q codeletion, achieving an accuracy of 75 ± 9% in predicting the IDH-mutation status, higher than using cMRI and multi-shell dMRI separately (both 70 ± 7%). Similar findings were observed for predicting the 1p/19q-codeletion status, with the accuracy from combining cMRI and multi-shell dMRI (72 ± 4%) higher than from each modality used alone (cMRI: 65 ± 6%; multi-shell dMRI: 66 ± 9%). These findings remain when we considered all 146 patients for predicting the IDH status (combined: 81 ± 5% accuracy; cMRI: 74 ± 5%; multi-shell dMRI: 73 ± 6%) and for the diagnosis of the three molecular subtypes according to WHO 2021 (combined: 60 ± 5%; cMRI: 57 ± 8%; multi-shell dMRI: 56 ± 7%). Together, these findings suggest that combining cMRI and multi-shell dMRI can offer higher accuracy than using each modality alone for predicting the IDH and 1p/19q status and in diagnosing the three molecular subtypes with DL-based models.

## 1. Introduction

The prognosis accuracy of diffuse gliomas, the most common tumours of the central nervous system (CNS), has been greatly improved in recent years with advances in molecular biomarkers. Two key molecular biomarkers are isocitrate dehydrogenase (IDH) genotype and epigenetic 1p/19q codeletion. Since 2016, the presence or absence of IDH-mutation and of 1p/19q codeletion [1] have been integrated into the WHO classification of adult-type gliomas [2]. IDH-mutant gliomas have a less poor prognosis and tend to occur in up to 80% of patients with lower grades gliomas (LGG, i.e., WHO-2 or -3) and in patients with secondary glioblastomas (GBM) [3]. In contrast, IDH-wildtype gliomas have worse prognosis and include primary GBMs and a minority of LGG-mimicking gliomas. The 1p/19q codeletion is a pathognomonic biomarker that defines oligodendrogliomas, a distinct glioma subtype, that is likely to respond better to chemotherapy [4].

The importance of molecular biomarkers has grown further in the fifth edition of the WHO classification of CNS tumours (WHO CNS5) released in 2021 [5]. In this update, adult-type gliomas now include three subtypes: “oligodendroglioma, IDH-mutant, 1p/19q codeleted”, “astrocytoma, IDH-mutant, uncodeleted”, and “glioblastoma, IDH-wildtype”. Oligodendrogliomas are associated with a better prognosis and a median overall survival (OS) of 8 years. Astrocytomas show an intermediate OS of 5–8 years. Glioblastomas are prone to early recurrence with short progression free survival (PFS) and OS < 2 years [6]. Of note, the subtype “glioblastoma, IDH-wildtype” in WHO CNS5 also includes the rare group of IDH-wildtype gliomas mimicking LGG, hereafter denoted as molecular GBM.

The demonstrated value of molecular biomarkers for prognosis motivates the development of non-invasive techniques that can infer molecular biomarkers from conventional MRI (cMRI) commonly used for diagnosis and treatment of CNS tumours. A few investigators have shown that cMRI features help predict the IDH status [7,8], perform more accurate prognoses, and enable the personalized treatment [9,10] of patients with gliomas. Several studies highlighted the value of post-contrast imaging in identifying patients with IDH-wildtype gliomas. However, tumour enhancement is inadequate as a discriminative feature, since it is observed also in up to 50% of patients with IDH-mutant gliomas with 1p/19q codeletion [11]. Moreover, a minority of IDH-wildtype tumours may not enhance, in particular, those with histopathological features mimicking LGGs [12,13,14].

Improvements to cMRI can be made with more advanced MRI techniques, such as diffusion MRI (dMRI), which provides measurements that inform tumour features at the micron scale. In particular, dMRI can be used to infer apparent diffusivity, which is usually higher in IDH-mutant than IDH-wildtype gliomas due to lower cellular density and increased interstitial edema. In 2021, Yan et al. showed that incorporating radiomic signatures from post-contrast T1-weighted (cMRI) and apparent diffusion coefficient (ADC) (derived from dMRI) achieved an accuracy of 82% for predicting the IDH status [15]. Other investigators have used diffusion tensor imaging (DTI) and multiple diffusion metrics (i.e., mean diffusivity (MD) and fractional anisotropy (FA)) for the subtyping of gliomas [16,17,18], 1p/19q codeletion status [16,19], and their correlation with therapy response and patient outcome [20,21].

The studies above are limited to the use of a single diffusion-weighting factor (b-value). These single-shell approaches can be improved when the appropriate instrumentation is available to include additional and significantly higher b-values. Such multi-shell approaches allow for the use of more informative diffusion models. Multi-shell dMRI enables, for example, diffusion kurtosis imaging (DKI), which provides additional metrics such as kurtosis anisotropy (KA) and mean kurtosis (MK). They are useful for predicting the IDH status, because higher tumour cell density results in higher kurtosis values [22]. Another example is neurite orientation dispersion and density imaging (NODDI), which uses a multi-compartment biophysical diffusion model. NODDI improves specificity to brain microstructure by taking into account, in a single voxel, the models for water diffusion in three different compartments: intracellular, extracellular, and cerebrospinal fluid [23]. Figini et al. showed that molecular GBMs present with a significant higher volumetric fraction of the intracellular compartment (fR), higher KA, and lower MD compared with IDH-mutant gliomas [24]. Taken together, these studies indicate that multi-shell dMRI carries complementary information not conveyed by cMRI or single-shell dMRI and could help identify the IDH and 1p/19q status of patients with gliomas.

Recent progress in deep learning models and techniques may provide powerful solutions to determine the molecular subtypes of gliomas using automatic learning of features directly from histopathology images [25,26] and, noninvasively, also from magnetic resonance images [27,28,29,30]. In particular, Residual Network (ResNet) architectures (ResNet34, ResNet50, ResNet101, and ResNet152) have been used for glioma segmentation, classification, and molecular subtype identification [31,32,33,34,35,36]. The extensive use of ResNet in the literature to accomplish several tasks in glioma research and other fields is due to the presence of shortcuts (residual connections) among the layers of the network, which helped to address the problem of vanishing gradients and training accuracy saturation compared to other neural network architectures. As a consequence, this has allowed the network depth to be increased while having faster training and higher accuracy. However, studies aiming to predict molecular subtypes of gliomas with MRI using ResNet or other deep learning algorithms have been based on cMRI alone or limited by the exclusive use of an ADC map [29,37]. To date the potential of adding microstructural features derived from advanced dMRI to deep learning models has yet to be explored.

In this study, we investigated whether adding multi-shell dMRI metrics to cMRI will improve the predictive performance of ResNet compared to using only cMRI, or only multi-shell dMRI. We trained several ResNet models based on multi-shell dMRI and cMRI, either alone or in combination, and compared their performances in different tasks. We hypothesize that using a combination of image features extracted by ResNet from multi-shell dMRI and cMRI may result in improved glioma molecular subtype assessment. This potential improvement was evaluated in two rounds of analysis to specifically understand if it was influenced by considering the LGG-mimicking molecular GBMs (1) alone or (2) together with IDH-wildtype GBMs.

## 2. Materials and Methods

### 2.1. Experimental Design

We investigated the network performances in three classification tasks that are the most clinically relevant and frequently investigated in neuro-oncological applications related to gliomas: (i) IDH-mutant versus IDH-wildtype; (ii) 1p/19q codeleted versus 1p/19q non-codeleted; and (iii) three molecular subtypes according to WHO CNS5 (IDH-mutant 1p/19q-codeleted oligodendroglioma, IDH-mutant astrocytoma, and IDH-wildtype glioblastoma). As part of a first round of analysis, task (i) and (ii) were conducted in patients with LGG as defined by the 2016 WHO classification, to exclude the effect of GBMs. Then, in the second round of analysis, tasks (i) and (iii) were performed considering the entire set of patients with low- and high-grade gliomas. This strategy will also help to compare our results with prior studies using either one of the two WHO classification schemes.

### 2.2. Dataset

The multimodal MRI and genetic data of patients with glioma were collected at the Humanitas Research Hospital, Rozzano (MI), Italy between April 2012 and November 2015, as part of a previous study [24]. A total of 146 patients with adult-type gliomas and no prior surgery were selected; the demographic patients data are summarized in Table 1. The first round of analysis was performed in 100 patients with LGG according to the 2016 WHO classification to predict the IDH mutation and 1p/19q codeletion without the interference of GBMs: 50 IDH-mutant and 1p/19q-codeleted oligodendrogliomas, 28 IDH-mutant and 1p/19q-uncodeleted astrocytomas, and 22 patients with IDH-wildtype gliomas mimicking LGG (molecular GBM). The second round was performed on 146 patients with three glioma subtypes according to WHO CNS5, to predict the IDH status and the molecular subtype: 62 IDH-wildtype glioblastomas (42.5%), 50 IDH-mutant and 1p/19q-codeleted oligodendrogliomas (34.2%), and 34 IDH-mutant and 1p/19q-uncodeleted astrocytomas (23.3%). This latter group included six IDH-mutant astrocytomas of grade 4 according to WHO CNS5.

### 2.3. Image Acquisition and Processing

The preoperative brain MR images were acquired with a 3-tesla MRI scanner (Magnetom Verio; Siemens, Erlangen, Germany). The cMRI protocol included T2-weighted (T2-w), pre-and post-contrast T1-weighted (T1-w and T1-cw, respectively), and FLAIR images. The multi-shell dMRI was acquired as follows: 8 volumes with b = 0 s/mm^2^, 20 volumes with b = 700 s/mm^2^, and 40 volumes with b = 2000 s/mm^2^. The quantitative maps derived from diffusion images encompassed MD from DTI, KA from DKI, and the fR from NODDI. The image acquisition and dMRI processing are described in greater detail in a previous study [24].

For each subject, the images from cMRI were co-registered with the corresponding b = 0 image used as a reference volume and by the rigid registration algorithm FLIRT using FSL tools (https://fsl.fmrib.ox.ac.uk/fsl/fslwiki/FLIRT, accessed on 21 December 2022). The mask of the tumours was extracted either using the HD-GLIO brain tumour segmentation tool (https://github.com/NeuroAI-HD/HD-GLIO, accessed on 21 December 2022) or, if the output of HD-GLIO was not satisfactory, by manually contouring in 3D-Slicer (http://www.slicer.org, accessed on 21 December 2022). The delineation of the tumour masks was validated by a neuroradiologist (A.B.). For each patient, the voxel values were normalized for each image of the cMRI protocol. Specifically, the mean intensity of the image was subtracted from the intensity value of each voxel, which was then divided by the standard deviation (i.e., Z-score normalization).

**Figure 1:**
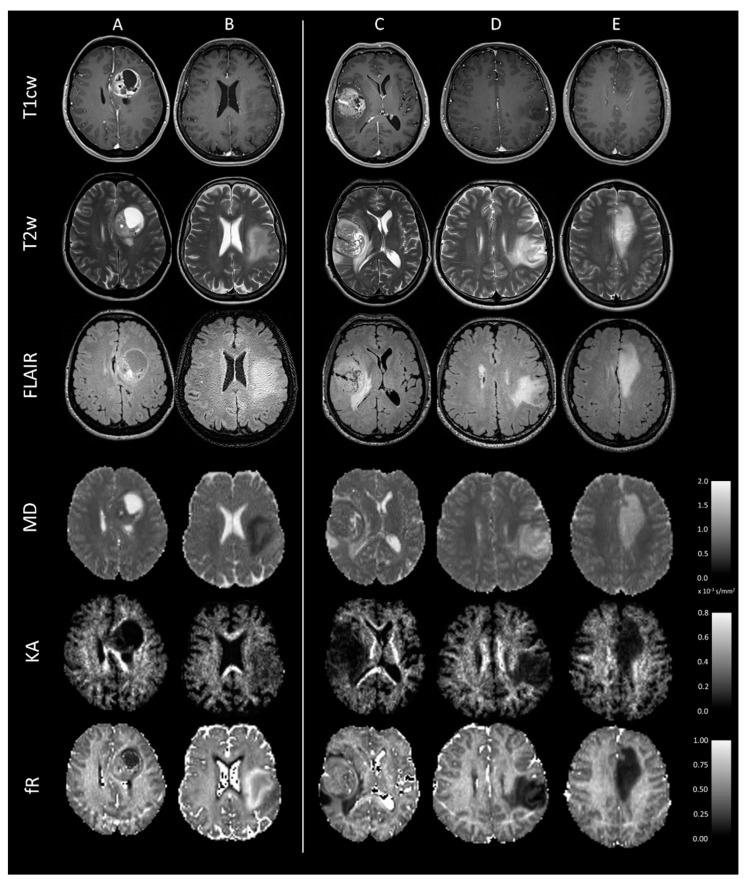
Axial T1cw, T2w, and FLAIR images and MD, KA, and fR maps at the level of the tumour are shown for five representative subjects with distinct molecular subtypes of adult-type gliomas. (**A**) Patient with IDH-wildtype glioblastoma: a necrotic central area is surrounded by a rim of enhancement following gadolinium injection with low MD and high KA and fR. This distinct radiologic appearance can help the network to distinguish this subtype from those with IDH-mutation. (**B**) Patient with molecular GBM: lack of enhancement and necrosis, but with low MD and high KA and fR. (**C**) Patient classified as grade 4 IDH-mutant astrocytoma according to WHO CNS5: radiological characteristics similar to an IDH-wildtype glioblastoma. (**D**) Patient with IDH-mutant astrocytoma: lack of enhancement and necrosis, with high MD and low KA and fR. (**E**) Oligodendroglioma with IDH-mutation and 1p/19q codeletion: lack or subtle contrast enhancement with high MD and low KA and fR. T1cw = post-contrast T1-weighted; T2w = T2-weighted; MD = mean diffusivity; KA = kurtosis anisotropy; fR = restricted fraction; GBM = glioblastoma; and WHO CNS5 = fifth edition of the WHO classification of the central nervous system tumours.

The MR images and diffusion metrics maps of representative cases of five molecular subtypes are illustrated in Figure 1.

### 2.4. Input Data

**Figure 2:**
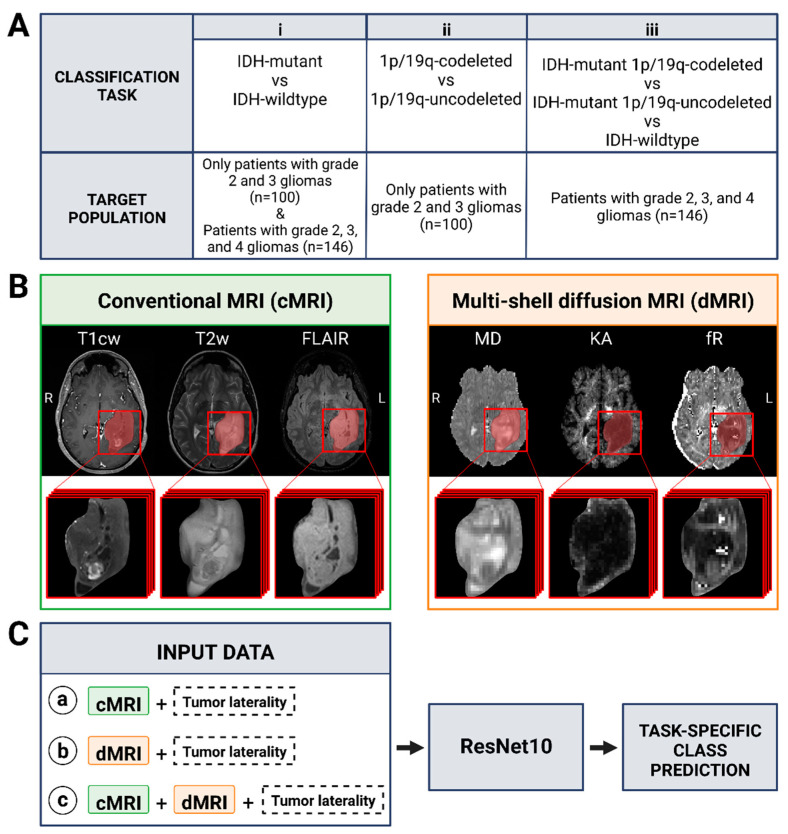
(**A**) Classification tasks considered in this study. Task (i) was performed in the entire set of patients included in the study (with WHO grade 2, 3, and 4 gliomas) and in the subset of those with lower-grades (2 and 3) gliomas. Task (ii) was performed only in the subset of patients with lower-grades gliomas. Task (iii) was performed only in the entire set of patients included in the study. (**B**) Representative conventional and diffusion MR images of a 23-year-old female patient with IDH-mutant and 1p/19q-codeleted oligodendroglioma. The tumour volumes (in red) of each image, obtained after semiautomatic segmentation, were subsequently used as input for the models. (**C**) Models developed for each task. They differ for the set of images used as input: (a) conventional MRI with tumour laterality (left or right brain hemisphere); (b) diffusion MRI with tumour laterality; (c) conventional and diffusion MRI with tumour laterality. The architecture of the residual network (ResNet10) used to provide the task-specific class prediction was the same. This figure was created with BioRender.com, accessed on 21 December 2022.

The networks were designed and trained based on three input sets: (1) 3D image patches extracted from normalized cMRI (T1-cw, T2-w, FLAIR) centred on tumour segmentation, (2) the corresponding 3D patches of quantitative maps derived from multi-shell dMRI (MD, KA, fR), and (3) the combination of cMRI and multi-shell dMRI inputs (Figure 2). In particular, the multi-shell dMRI inputs were selected on the basis of previous results [21] that had shown MD, KA, and fR to have the highest significant differences between molecular subtypes; other diffusion metrics were not considered. The input patches were chosen to be of the size 64 × 64 × 15, which was large enough to fit all the tumours. The laterality of tumours (located on the left or right brain hemisphere) was included as an additional input.

### 2.5. ResNet Architecture Description and Model Development

The ResNet10 model built into the PyTorch library (https://pytorch.org, accessed on 21 December 2022) was adapted for this study. The ResNet10 model consisted of 10 3D convolutional layers, batch-normalization, followed by average max-pooling and a fully connected layer. The rectified linear unit (ReLU) activation function was used in the convolutional layers. The Softmax activation function was used in the fully connected layer and cross entropy was used as loss function. The model was trained end-to-end using an adaptive moment estimation optimizer (Adam) with a batch size of 32 and maximal epochs of 100. An initial learning rate of 10^−6^, a momentum of 0.9, and a weight decay of 0.1 were used. A learning rate scheduler was applied to allow reducing the learning rate with gamma of 0.1 and patience of 10 based on the validation results. This model trained on the entire dataset for IDH prediction was repurposed as the starting point for the other models. This is an optimization technique called transfer learning: it is used for saving time and achieving better performance on small datasets and it works well if the features learnt in the first task are suitable for the new task.

To mitigate the limitations of the modest sample size of our patient cohort and to reduce overfitting, data augmentation was applied during model training. Data augmentation included vertical flipping, horizontal flipping, translation, rotation, and addition of Gaussian noise to the 3D input data. Moreover, the oversampling of the minority class was performed during training to mitigate the class imbalance of the training set.

**Figure 3:**
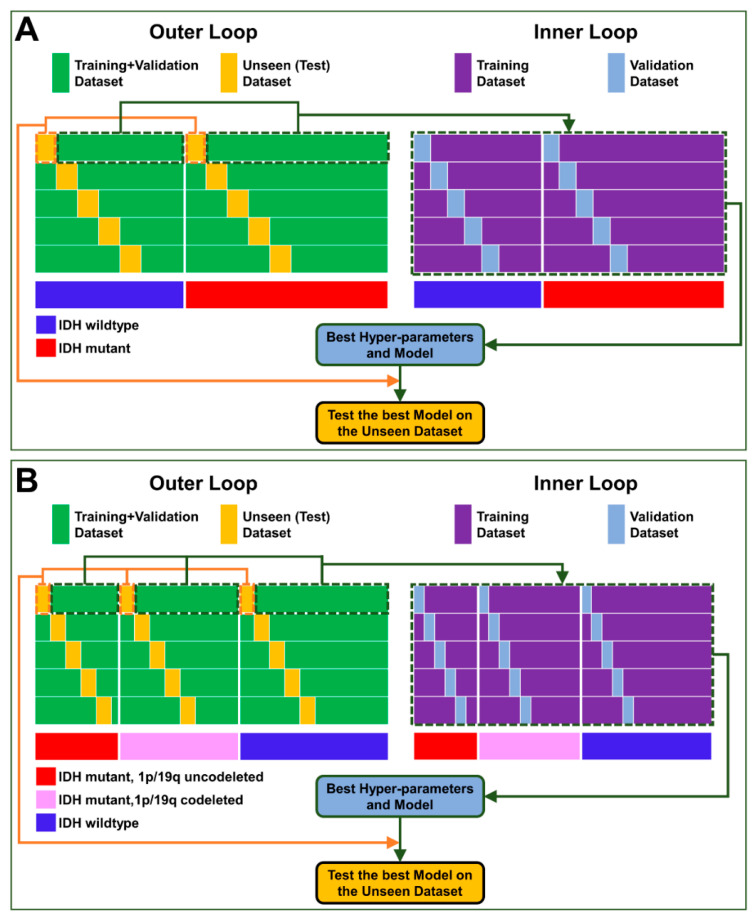
Scheme illustrating the nested cross-validation procedure for developing the network for (**A**) a two-group prediction task (as an illustrative example, IDH status prediction) and (**B**) a three-group prediction task (i.e., diagnosis of the three molecular subtypes according to WHO 2021 classification). (Outer loop) Splitting the dataset into unseen test dataset (orange) and training–validation dataset (green) with a 5-fold cross-validation. (Inner loop) Splitting training–validation dataset into training (violet) and validation (blue) datasets with a 5-fold cross-validation for feature extraction and hyperparameter optimization. All splits were performed to have balanced classes in the validation and in the test sets. At the end of the outer loop, the results of the models tested on the 5 unseen test sets were averaged together to report the final classification results.

To reliably evaluate the performance of each network, we used a nested cross-validation comprising an outer loop with 5-fold cross-validation to evaluate the classification performance and an inner loop with 5-fold cross-validation to tune the model hyperparameters and find the best features (Figure 3). In the outer loop, the held-out test sets were chosen that preserved the class balance (Table 2). In the inner loop, the validation sets were chosen so that they have the same (balanced) class distribution as the test sets (Table 2). The model was trained in the inner loop on each of the 5 training sets and validated on the corresponding validation set: the model that achieved maximum validation accuracy and minimum loss was selected. Then, the selected model was tested on the corresponding held-out unseen set in the outer loop. The results of the 5 runs of model-testing were averaged together to evaluate the final classification performance of the network. The validation and unseen test sets remained the same for all different modality combinations in each classification task. Only for the second round of analysis, we balanced molecular GBMs and IDH-wildtype GBMs within the IDH-wildtype class for each validation and test set.

### 2.6. Evaluation of the Classification Performance

The classification performance of each network was evaluated by measuring the individual class sensitivity of each group, as well as overall through the following metrics: accuracy, average precision (i.e., average of the positive predictive values of the groups), and the Matthews correlation coefficient (MCC). The MCC is a robust metric that summarizes the classifier performance in a single value ranging from −1 (total disagreement) to +1 (perfect classification), with a value of 0 indicating random prediction; it is particularly useful when positive and negative cases are of equal importance [38].

## 3. Results

In this section, we illustrate the classification performances of the networks obtained in the two rounds of analysis.

### 3.1. IDH and 1p/19q Status Prediction in Lower Grade Gliomas

In the first round of analysis on 100 patients with LGG, the model combining multi-shell dMRI and cMRI achieved an average accuracy of 75 ± 9% for IDH status (Table 3) and 72 ± 4% for 1p/19q status prediction (Table 4). The cMRI-only and multi-shell dMRI-only networks achieved lower classification accuracies in predicting the IDH status (both 70 ± 7%) and the 1p/19q codeletion status (65 ± 6% and 66 ± 9%, respectively). Besides accuracy, the average precision and the MCC coherently also indicated that the model combining cMRI and multi-shell dMRI achieved the best performance in patients with LGGs.

The detailed molecular subtype prediction results in LGGs are also shown in Table 3 and Table 4. As for the tasks related to IDH-mutation prediction, all the networks had higher sensitivity for the IDH-mutant group and had lower performances in correctly identifying the IDH-wildtype gliomas. In the task related to the 1p/19q-codeletion status prediction, the networks based on dMRI alone or in combination with cMRI had higher performances in correctly identifying the 1p/19q-uncodeleted gliomas.

### 3.2. IDH Status and Three Molecular Subtypes Prediction in All Glioma Grades

**Figure 4:**
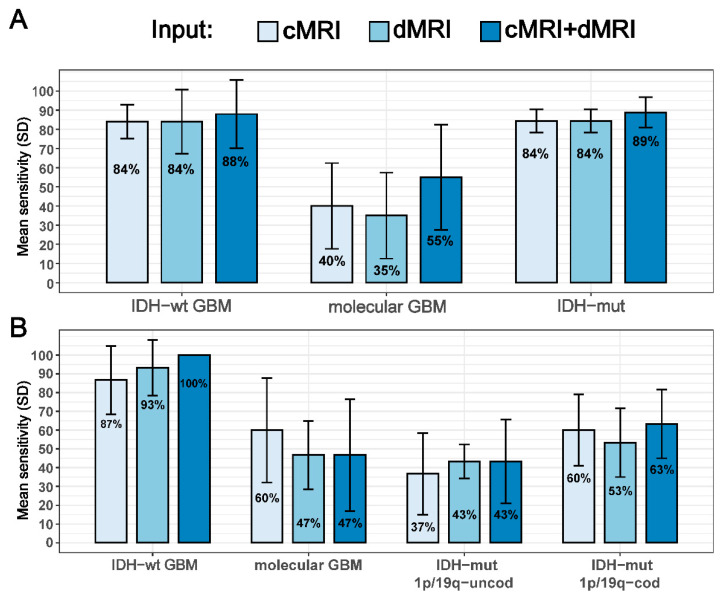
Individual molecular subtype prediction performance of the networks using different MR images as inputs for two tasks in the second round of analysis that included glioblastomas: (**A**) IDH status prediction and (**B**) three-group molecular subtype prediction. The numbers within the coloured bars are the average sensitivities over the five test sets in the outer loop and the vertical black bars represent the corresponding standard deviations. cMRI = conventional MRI; dMRI = diffusion MRI; IDH-wt = IDH-wildtype; GBM = glioblastoma; IDH-mut = IDH-mutant; 1p/19q-cod = 1p/19q-codeleted; 1p/19q-uncod = 1p/19q-uncodeleted.

The second round of analysis was performed on the whole cohort of 146 patients with low and high grade adult-type gliomas. For IDH status prediction, the ResNet model combining multi-shell dMRI and cMRI achieved an average accuracy of 81% ± 5%, higher than the other two models. The cMRI-based and multi-shell dMRI-based networks individually achieved an average accuracy of 74 ± 5% and 73 ± 6%, respectively (Table 5). Besides accuracy, precision and MCC also indicated that the model combining cMRI and multi-shell dMRI achieved the best performance. Among the patients with IDH-wildtype glioma, all the networks had high sensitivity in the test set for grade 4 (GBM), which were correctly diagnosed in most of the cases (cMRI+dMRI: 88%; cMRI: 84%; dMRI: 84%) (Figure 4A). However, lower sensitivities were obtained for molecular GBMs (cMRI+dMRI: 55%; cMRI: 40%; dMRI: 35%), which were often incorrectly diagnosed as IDH-mutant gliomas. The ResNet model combining multi-shell dMRI and cMRI had the highest sensitivities among the networks for both IDH-wildtype subgroups (Figure 4A), as well as for IDH-mutant gliomas (cMRI+dMRI: 89%; cMRI: 84%; dMRI: 84%) (Table 5 and Figure 4A).

For the direct prediction of the three molecular subtypes, the ResNet model had an overall accuracy of 60 ± 5% when cMRI and multi-shell dMRI were combined. Both the cMRI-based and multi-shell dMRI-based networks had lower accuracies: 57 ± 8% and 56 ± 7%, respectively (Table 6). Besides accuracy, precision and MCC indicated that the model combining cMRI and multi-shell dMRI achieved the best performance also for this task. Of note, the patients with grade 4 IDH-wildtype GBM were all diagnosed correctly in the test set when multi-shell dMRI was used in combination with cMRI and with lower sensitivities when cMRI and dMRI were used alone (Figure 4B). In contrast, low sensitivities were obtained for molecular GBMs (cMRI+dMRI: 47%; cMRI: 60%; dMRI: 47%), which were often incorrectly classified as IDH-mutant gliomas (Figure 4B). As for the two subgroups with IDH-mutant glioma, all the networks had higher sensitivities for the IDH-mutant 1p/19q-codeleted oligodendroglioma than the IDH-mutant uncodeleted astrocytoma subtype (Table 6 and Figure 4B).

## 4. Discussion

To the best of our knowledge, this is the first time that a deep learning-based model using a combination of conventional and multi-shell diffusion MRI has been designed and validated to classify gliomas. The classification accuracies were reliably estimated through a nested cross-validation procedure and fairly compared among networks by keeping the same training, validation, and held-out testing sets for all different combinations of modalities in every single classification task. Adding multi-shell dMRI to conventional MRI improved the prediction of IDH status, 1p/19q codeletion status, and diagnosis of the three molecular subtypes of adult-type gliomas according to WHO CNS5.

The results of this study provide additional evidence that deep learning models based on multi-modal MRI perform better than those adopting only one MRI modality. Specifically, by showing that the addition of advanced multi-shell dMRI sequences increased the molecular subtype diagnostic accuracy of deep-learning algorithms, we extend previous results that demonstrated a gain in accuracy only considering standard single-shell dMRI data (i.e., ADC maps). In particular, Cluceru et al. [37] performed a study in 384 patients with low- and high-grade glioma (including eight molecular GBMs) and showed that adding ADC maps to cMRI (i.e., T2-FLAIR and post-contrast T1w) in a deep convolutional neural network (namely VGG) improved the molecular subtype classification results from 82% to 86%. Another study performed by Matsui et al. [35] in 217 LGG patients (including 49 molecular GBMs) and using ResNet, achieved 69% accuracy by extracting multimodal features (i.e., cMRI including T1-w, T2-w, and FLAIR images, along with PET and CT images, but not diffusion imaging), higher than the accuracy obtained with cMRI alone (58%).

Our findings are in agreement with several prior studies showing that the diffusion metrics evaluated in this study (i.e., MD, KA, and fR) capture microstructural features that can predict IDH mutation and 1p/19q codeletion with good accuracy. The majority of prior studies employing dMRI for molecular subtype diagnosis in gliomas have used standard methods of ROI analysis [19,24,39,40,41,42,43,44,45,46,47,48,49], while a few used machine learning methods based on handcrafted (radiomic) features [15,50,51,52,53,54,55,56,57,58,59] and only one deep learning algorithms [37].

Deep-learning approaches are flexible in the choice of features, as they automatically learn discriminative high-level features directly from images, although they typically require more samples (i.e., subjects) than standard machine learning models. In contrast, machine learning methods based on radiomic features can work well also on relatively small sample sizes but are limited by the fact that features are pre-determined and subject to the personal choice of the researcher and meaningful features may be missed. Prior studies applying machine learning models based on radiomic features also found that multimodal datasets encompassing dMRI data may improve classification accuracies. In a study performed to predict the IDH status in 357 patients with adult-type glioma, Yan et al. [15] found that a model incorporating radiomic features extracted from post-contrast T1-w images and ADC maps led to an area under the curve of 0.884 for predicting IDH-mutation status, higher than that obtained with post-contrast MRI alone (0.869). Similarly, Kihira et al. [53] reported that combining radiomic features extracted from dMRI (i.e., DWI with b = 1000 and ADC maps) and cMRI (i.e., FLAIR and post-contrast T1-w images) improved the prediction of the IDH status from 76% (cMRI alone) to 79% (cMRI combined with ADC) accuracy in a cohort of 111 patients. In another study on 168 LGG patients, Park et al. [56] found that combining radiomic features derived from ADC and FA with those of conventional MRI reached 0.900 area under the curve, which is significantly higher than 0.835 obtained by using only radiomic features from conventional imaging.

Studies applying standard ROI analyses of ADC maps [42,44,45,46,48] and additional metrics derived from multi-shell dMRI [19,43,47,49] have also shown good accuracy in validation/test sets up to 86% for IDH mutation prediction. These studies have the advantage to provide direct biological interpretation of the results, while in contrast deep-learning studies have the reputation to be “black-boxes”. However, ROI analysis studies suffer from similar limitations of machine learning and radiomic-based studies, because they investigate only a small number of predefined features and they usually require more human intervention in several steps of the analysis than that required by deployed deep-learning models. The choice of the imaging features by an expert introduces biases and it is a major limitation to the reproducibility, the automation, and the degree of integration into clinical practice that can be reached.

The wide range of accuracy values obtained in the three tasks of our deep learning study deserve further attention, especially when we compare them with those of the aforementioned studies. The comparison of the results obtained by studies using different methods of analysis is always difficult and it may also depend on differences in the patient population. To investigate this issue further, we examined group sensitivities to determine which were the glioma subtypes more often misclassified by the network in the three tasks.

The first task was to predict the IDH status. In the most recent 2021 WHO classification, the subtype “IDH-wildtype glioblastoma” includes not only GBMs but also molecular GBMs, while in the 2016 WHO classification these tumours were classified as LGG. The diagnosis by means of MRI of these rare IDH-wildtype tumours would be very important for personalised medicine; however, it remains particularly challenging because they imitate IDH-mutant gliomas. Molecular GBMs exhibit clinical features similar to GBM with short PFS and OS but, viewed with histopathology and conventional MRI (Figure 1B), they show a relatively benign appearance resembling LGG, with a low proliferative index, low cellular density, and no necrosis nor enhancement after intravenous contrast injection. To better understand how the network performance was influenced by considering molecular GBM alone or together with IDH-wildtype GBM, we conducted two rounds of analysis. In the first round, we investigated the classification performances of our models in the subset of 100 patients with LGGs. In the second round, we considered all the glioma patients including GBMs. Not surprisingly, the accuracy of IDH status prediction was lower in the first than in the second round (75% vs. 81%). This stems from the exclusion of GBMs in the first round. In contrast to molecular GBM, IDH-wildtype GBMs can be easily distinguished from IDH-mutant gliomas by radiologists due to their remarkable intra-tumour heterogeneity with areas of necrosis, blood–brain barrier breakdown, and extensive perifocal edema (Figure 1A). These distinct radiologic features likely helped the network in the second round of analysis to find relevant MR image features able to correctly identify IDH-wildtype GBM with high sensitivity (up to 88%). In addition, the majority of our LGG patients, as expected, had the IDH mutation (~80%), therefore the deep learning network may have had difficulties to find relevant features for the rarer subgroup of molecular GBMs.

Indeed, all our networks had low performances in identifying patients with molecular GBMs, consequently reducing the sensitivity for the whole IDH-wildtype group and, with that, the overall accuracy. This important issue may have been overlooked in several previous studies, in part due to the low incidence of molecular GBMs in those studies. In contrast, the number of these patients was relatively large in our study. Moreover, the prediction performance of a model in the IDH-wildtype class depends on the proportion of IDH-wildtype GBMs and molecular GBMs. In our second round of analysis, we took into account this crucial aspect by maintaining the balance between molecular GBMs and IDH-wildtype GBM in test and validation sets. Of note, if the molecular GBM patients had not been included in that analysis, the accuracy of the best performing network in predicting IDH mutation would have increased from 81% to 88%.

The second task was to predict 1p/19q codeletion status: the network made more errors when identifying IDH-mutant (uncodeleted) astrocytomas than IDH-mutant (codeleted) oligodendrogliomas. Two critical issues may have resulted in lower accuracy for the former class. First, as suggested by Cluceru et al. [37], the power of deep learning models to discriminate the 1p/19q-codeletion status may be still limited. Second, similar to other authors that investigated 1p/19q status prediction, we included IDH-mutant and IDH-wildtype gliomas in the LGG cohort to replicate a real clinical scenario where no molecular information of the tumour is available before surgery. However, IDH-wildtype are different from IDH-mutant tumours and the 1p/19q-codeletion status has uncertain clinical significance for them. Therefore, their inclusion might have caused the identification of image features able to characterize and discriminate 1p/19q-codeletion status to be more difficult, thus decreasing the performance of the models.

The third task was to predict the three molecular glioma subtypes according to WHO CNS5. We obtained the highest accuracy when cMRI and multi-shell dMRI were combined (60% ± 5%), while the models using cMRI or multi-shell dMRI data alone had lower performances (56% ± 7% and 56% ± 6%, respectively). These values are significantly lower than those obtained in the previous two tasks because here we addressed a three-group and not a simpler two-group classification task, as was the case for IDH and 1p/19q status predictions. Once again, the molecular GBMs were diagnosed with lower sensitivity than IDH-wildtype GBMs, reducing the overall accuracy since for this task we also kept an equal sample size of molecular GBMs and IDH-wildtype GBM during validation and testing. Although our dataset was relatively small for a three-subtype classification, the network could identify the IDH-wildtype group (73%) with higher sensitivity than the IDH-mutant 1p/19q-codeleted oligodendrogliomas (63%) and IDH-mutant astrocytomas (43%). This result was not surprising because of the peculiar MRI features of GBMs as discussed above. Additionally, in this case, if we had not included patients with molecular GBM in the IDH-wildtype group, the overall accuracies would have increased from 60% to 69%. Cluceru et al. [37] conducted a three-group classification analysis and they obtained higher sensitivity for the IDH-wildtype group (95%) compared to IDH-mutant astrocytoma (89%) and IDH-mutant 1p/19q-codeleted oligodendroglioma (60%). The authors recruited 143 GBMs and only eight low grades in the IDH-wildtype cohort: this difference in the population may explain the difference in accuracy between their study and ours. These results emphasize that the distribution of the different glioma subtypes in a study cohort is important and it can have a profound effect on the accuracy of the study. Our study confirms that molecular GBM is the most difficult class of glioma to predict. Deep learning methods as well as machine learning and ROI-analysis based methods appear to underperform when molecular GBMs are represented in larger numbers in the cohort.

Some limitations of this study should be acknowledged. First, this study was retrospective and the data were collected in a single centre. A larger multi-centre study would be required to assess the generalizability of our findings. Second, we had a relatively modest and, similar to the majority of studies in this field, imbalanced dataset. Imbalanced classification poses a challenge for predictive modelling and results in poor predictive performance, specifically for the minority class because the model may focus on learning the features of the majority class and neglect the few samples of the minority class. Hence, the accuracy and precision can be biased due to the presence of imbalanced classes in the training and test sets, contributing to different performances for different studies as discussed above. To mitigate this issue, we applied the oversampling technique for the training dataset and kept the balance between class distribution in validation and test sets. Third, we focused on MD, KA, and fR as multi-shell dMRI-derived input for all the classification tasks based on the results of a previous study [24] that had shown these indicators of microstructural features were the most useful to differentiate the molecular subtypes. However, different tasks might have different sensitivity to different diffusion metrics. In future studies, a comprehensive evaluation of other diffusion models such as IVIM [60], VERDICT [61], or DIVIDE [62] should be tested.

## 5. Conclusions

The preoperative diagnosis of molecular subtypes with deep learning is feasible and may help surgeons and oncologists to make treatment decisions, including surgery, radiotherapy, chemotherapy, or immunotherapy. We demonstrated that a deep learning-based model using a combination of conventional and multi-shell diffusion MRI data provided more accurate predictions of IDH, 1p/19q status and diagnosis of the three molecular subtypes of adult-type gliomas than a model based only on one modality. The accuracy of the models in three tasks with two rounds of analysis depended on the size of the population and the appropriate balance among the number of patients included in each subtype group. The differences in class accuracies outlined in this study emphasize the importance of using a balanced set of patients with all glioma subtypes equally represented during model training and testing. Future studies on glioma molecular subtype prediction should take into consideration this important aspect in the experimental design.

## Figures and Tables

**Table 1:** Demographic and clinical data of the patient cohorts.

Clinical Characteristics	Values
Number of subjects, N (%)	146 (100)
Age, mean (range) years	46.2 (14–77)
Sex	
Male, N (%)	88 (60)
Female, N (%)	58 (40)
Hemisphere	
Left, N (%)	95 (65)
Right, N (%)	45 (31)
Bilateral, N (%)	6 (4)
WHO grade	
2, N (%)	50 (34)
3, N (%)	50 (34)
4, N (%)	46 (32)
Molecular subtype	
Grade 2–3 diffuse astrocytoma, IDH-mutant, N (%)	28 (19.2)
Oligodendroglioma, IDH-mutant, 1p/19q codeleted, N (%)	50 (34.2)
Molecular glioblastoma, IDH-wildtype, N (%)	22 (15)
Grade 4 diffuse astrocytoma, IDH-mutant, N (%)	6 (4.1)
Glioblastoma, IDH-wildtype, N (%)	40 (27.5)

IDH = isocitrate dehydrogenase.

**Table 2:** Class distribution in training, validation, and test sets for each classification task.

Target Population	Classification Task	Training ^1^	Validation ^1^	Test ^2^
Only patients with grade 2 and 3 gliomas (*n* = 100)	IDH-mutant (*n* = 78) vs. IDH-wildtype (*n* = 22)	*n* = 72 vs. *n* = 16	*n* = 2 vs. *n* = 2	*n* = 4 vs. *n* = 4
	1p/19q-codeleted (*n* = 50) vs. 1p/19q-uncodeleted (*n* = 50)	*n* = 41 vs. *n* = 41	*n* = 3 vs. *n* = 3	*n* = 6 vs. *n* = 6
Patients with grade 2, 3, and 4 gliomas (*n* = 146)	IDH-mutant (*n* = 84) vs. IDH-wildtype (*n* = 62)	*n* = 71 vs. *n* = 49	*n* = 4 vs. *n* = 4	*n* = 9 vs. *n* = 9
	IDH-mutant and 1p/19q-codeleted (*n* = 50) vs. IDH-mutant and 1p/19q-uncodeleted (*n* = 34) vs. IDH-wildtype (*n* = 62)	*n* = 40 vs. *n* = 24 vs. *n* = 52	*n* = 4 vs. *n* = 4 vs. *n* = 4	*n* = 6 vs. *n* = 6 vs. *n* = 6

^1^ The reported numbers of patients in training and validation tests refer to one split within an inner loop.^2^ The reported numbers of patients in test sets refer to one split within the outer loop.

**Table 3:** IDH status classification performance in LGGs (WHO 2016).

Classification Metrics	cMRI	Multi-Shell dMRI	cMRI and Multi-Shell dMRI
**Overall performance**			
Accuracy	70% ± 7%	70% ± 7%	75% ± 9%
Precision ^1^	0.81 ± 0.03	0.78 ± 0.03	0.82 ± 0.06
MCC	0.50 ± 0.11	0.47 ± 0.09	0.56 ± 0.14
**Individual group sensitivity**			
IDH mutant	100% ± 0%	75% ± 31%	95% ± 11%
IDH wildtype	40% ± 14%	65% ± 28%	55% ± 21%

Classification performance of ResNet on the unseen test sets with different MRI inputs to predict IDH-mutation versus IDH-wildtype status in lower-grade gliomas according to the 2016 WHO classification. Data are reported as average ± standard deviation over the five test sets in the outer loop. Overall, data of 40% (40 out of 100) LGGs patients were tested as unseen sets, covering almost all IDH-wildtype lower-grades gliomas in our cohort. ^1^ Computed as the macro-average of the precisions for the IDH-mutant group and the IDH-wildtype group. IDH = isocitrate dehydrogenase; LGG = lower-grades glioma; cMRI = conventional MRI; dMRI = diffusion MRI; MCC = Matthews correlation coefficient.

**Table 4:** 1p/19q status classification performance in LGGs (WHO 2016).

Classification Metrics	cMRI	Multi-Shell dMRI	cMRI and Multi-Shell dMRI
**Overall performance**			
Accuracy	65% ± 6%	66% ± 9%	72% ± 4%
Precision ^1^	0.66 ± 0.06	0.73 ± 0.10	0.75 ± 0.05
MCC	0.30 ± 0.10	0.38 ± 0.20	0.45 ± 0.08
**Individual group sensitivity**			
1p/19q codeleted	70% ± 10%	60% ± 10%	60% ± 10%
1p/19q uncodeleted	61% ± 20%	70% ± 20%	83% ± 10%

Classification performance of ResNet on the unseen test sets with different MRI inputs to predict 1p/19q codeletion versus 1p/19q non-codeletion (IDH-mutant or IDH-wildtype) in LGGs according to the 2016 WHO classification. Data are reported as average ± standard deviation over the five test sets in the outer loop. Overall, data of 60% (60 out of 100) LGGs patients were tested as unseen set. ^1^ Computed as the macro-average of the precisions for the IDH-mutant group and the IDH-wildtype group. LGG = lower-grades glioma; cMRI = conventional MRI; dMRI = diffusion MRI; MCC = Matthews correlation coefficient.

**Table 5:** IDH status classification performances in adult-type gliomas (WHO CNS5).

Classification Metrics	cMRI	Multi-Shell dMRI	cMRI and Multi-Shell dMRI
**Overall performance**			
Accuracy	74 ± 5%	73 ± 6%	81 ± 5%
Precision ^1^	0.77 ± 0.04	0.77 ± 0.09	0.83 ± 0.04
MCC	0.52 ± 0.08	0.52 ± 0.18	0.64 ± 0.06
**Individual group sensitivity**			
IDH mutant	84 ± 6%	84 ± 6%	89 ± 8%
IDH wildtype	64 ± 9%	62 ± 6%	73 ± 13%

Classification performance of ResNet on the unseen test sets with different MRI inputs to diagnose IDH mutation status on the whole cohort of 146 patients with low- and high-grade gliomas. Data are reported as average ± standard deviation over the five test sets in the outer loop. ^1^ Computed as the macro-average of the precisions for the IDH-mutant group and the IDH-wildtype group. IDH = isocitrate dehydrogenase; cMRI = conventional MRI; dMRI = diffusion MRI; MCC = Matthews correlation coefficient.

**Table 6:** Subtype classification performances in adult-type gliomas (WHO CNS5).

Classification Metrics	cMRI	Multi-Shell dMRI	cMRI and Multi-Shell dMRI
**Overall performance**			
Accuracy	57 ± 8%	56 ± 7%	60 ± 5%
Precision ^1^	0.60 ± 0.14	0.59 ± 0.07	0.65 ± 0.05
MCC	0.37 ± 0.14	0.35 ± 0.10	0.43 ± 0.06
**Individual group sensitivity**			
IDH-mutant 1p/19q-codeleted	60 ± 19%	53 ± 18%	63 ± 18%
IDH-mutant 1p/19q-uncodeleted	37 ± 22%	43 ± 9%	43 ± 22%
IDH wildtype	73 ± 19%	70 ± 7%	73 ± 15%

Classification performance of ResNet on the test sets with different MRI inputs to diagnose glioma molecular subtype according to WHO CNS5 on the whole cohort of 146 patients. Data are reported as average ± standard deviation over the five test sets in the outer loop. ^1^ Computed as the macro-average of the precisions for the three groups. WHO CNS5 = fifth edition of the WHO classification of the central nervous system tumours; IDH = isocitrate dehydrogenase; cMRI = conventional MRI; dMRI = diffusion MRI; MCC = Matthews correlation coefficient.

## Data Availability

The source code with the trained model is available on GitHub (https://github.com/GKaramiMP/ResNetModel, accessed on 21 December 2022). The dataset generated during and/or analysed during the current study is not publicly available due to the clinical and confidential nature of the material but can be made available from the corresponding author on reasonable request.
